# Supplementary material for: ColabFold: making protein folding accessible to all
Source: Nat Methods. 2022 May 30;19(6):679–82. doi: 10.1038/s41592-022-01488-1 (PMC9184281; doi:10.1038/s41592-022-01488-1)
Supplement: Supplementary file 2 — Reporting Summary [file 41592_2022_1488_MOESM2_ESM.pdf]

nature research

Corresponding author(s): Milot Mirdita, Sergey Ovchinnikov, Martin Steinegger

Last updated by author(s): Apr 1, 2022

Reporting Summary

Nature Research wishes to improve the reproducibility of the work that we publish. This form provides structure for consistency and transparency in reporting. For further information on Nature Research policies, see our Editorial Policies and the Editorial Policy Checklist.

Statistics

For all statistical analyses, confirm that the following items are present in the figure legend, table legend, main text, or Methods section.

n/a	Confirmed	
☐	☒	The exact sample size (*n*) for each experimental group/condition, given as a discrete number and unit of measurement
☒	☐	A statement on whether measurements were taken from distinct samples or whether the same sample was measured repeatedly
☒	☐	The statistical test(s) used AND whether they are one- or two-sided<br>*Only common tests should be described solely by name; describe more complex techniques in the Methods section.*
☒	☐	A description of all covariates tested
☒	☐	A description of any assumptions or corrections, such as tests of normality and adjustment for multiple comparisons
☒	☐	A full description of the statistical parameters including central tendency (e.g. means) or other basic estimates (e.g. regression coefficient) AND variation (e.g. standard deviation) or associated estimates of uncertainty (e.g. confidence intervals)
☒	☐	For null hypothesis testing, the test statistic (e.g. F, t, r) with confidence intervals, effect sizes, degrees of freedom and P value noted<br>*Give P values as exact values whenever suitable.*
☒	☐	For Bayesian analysis, information on the choice of priors and Markov chain Monte Carlo settings
☒	☐	For hierarchical and complex designs, identification of the appropriate level for tests and full reporting of outcomes
☒	☐	Estimates of effect sizes (e.g. Cohen's d, Pearson's r), indicating how they were calculated

Our web collection on statistics for biologists contains articles on many of the points above.

Software and code

Policy information about availability of computer code

Data collection	ColabFold is free open-source software (MIT) and available at https://github.com/sokrypton/ColabFold. A locally installable version (MIT) is available at https://github.com/YoshitakaMo/localcolabfold. The ColabFold development version shown in this manuscript is available at https://github.com/konstin/ColabFold. This version will shortly be integrated into the main repository. The ColabFold server components are free open-source software (GPLv3) and available at https://github.com/soedinglab/mmseqs2-app. MMseqs2 is free open-source software (GPLv3) and available at https://mmseqs.com. The ColabFold databases are available at https://colabfold.mmseqs.com under CC-BY 4.0 license.
Data analysis	Benchmark data analysis and visualization was done with R/4.1.1, ggplot/3.3.5, cowplot/1.1.1, lubridate/1.7.10. ColabFold generated plots were made using matplotlib/3.1.3. TM-score analysis was done with TMalign/2021/02/24 and DockQ/3735c16.<br>Data was generated with the following software: MMseqs2 (github commit edb822), Colabfold (github commit 45ad0e9), RoseTTAFold (github commit fcf9125), HHblits v3.3.0 and AlphaFold2 v2.1.1

For manuscripts utilizing custom algorithms or software that are central to the research but not yet described in published literature, software must be made available to editors and reviewers. We strongly encourage code deposition in a community repository (e.g. GitHub). See the Nature Research guidelines for submitting code & software for further information.

Data

Policy information about availability of data

All manuscripts must include a data availability statement. This statement should provide the following information, where applicable:

- Accession codes, unique identifiers, or web links for publicly available datasets
- A list of figures that have associated raw data
- A description of any restrictions on data availability

Code Availability
ColabFold is free open-source software (MIT) and available at github.com/sokrypton/ColabFold.
A locally installable version is available at github.com/YoshitakaMo/localcolabfold.
The ColabFold development version shown in this manuscript is available at github.com/konstin/ColabFold.
The ColabFold server components are free open-source software (GPLv3) and available at github.com/soedinglab/mmseqs2-app.
MMseqs2 is free open-source software (GPLv3) and available at mmseqs.com.

Data Availability
ColabFold databases are freely (CC-BY-SA 4.0) available at colabfold.mmseqs.com.
MSAs and structures produced during benchmarking:
wwwuser.gwdg.de/~compbiol/colabfold/manuscript
Input databases used for building ColabFold databases:
UniRef30: uniclust.mmseqs.com
BFD: bfd.mmseqs.com
MGnify:ftp.ebi.ac.uk/pub/databases/metagenomics/peptide_database/2019_05
PDB70: wwwuser.gwdg.de/~compbiol/data/hhsuite/databases/hhsuite_dbs
MetaEuk: wwwuser.gwdg.de/~compbiol/metaeuk/2019_11/MetaEuk_preds_Tara_vs_euk_profiles_uniqs.fas.gz
SMAG: www.genoscope.cns.fr/tara/localdata/data/SMAGs-v1/SMAGs_v1_concat.faa.tar.gz
TOPAZ: osf.io/gm564
MGV: portal.nersc.gov/MGV/MGV_v1.0_2021_07_08/mgv_proteins.faa
GPD: ftp.ebi.ac.uk/pub/databases/metagenomics/genome_sets/gut_phage_database/GPD_proteome.faa
Further datasets used for benchmarking ColabFold:
PFAM (Pfam-A.seed.gz & Pfam-A.full.gz): ftp.ebi.ac.uk/pub/databases/Pfam/releases/Pfam34.0
textit{M. jannaschii proteome: uniprot.org/proteomes/UP000000805 ftp.ebi.ac.uk/pub/databases/alphafold/v1/UP000000805_243232_METJA_v1.tar

Field-specific reporting

Please select the one below that is the best fit for your research. If you are not sure, read the appropriate sections before making your selection.

☒ Life sciences ☐ Behavioural & social sciences ☐ Ecological, evolutionary & environmental sciences

For a reference copy of the document with all sections, see nature.com/documents/nr-reporting-summary-flat.pdf

Life sciences study design

All studies must disclose on these points even when the disclosure is negative.

Sample size	ColabFold was evaluated on all CASP14 targets for single-chain predictions. For complex predictions, ColabFold was evaluated on the publicly available ClusPro dataset. We do not compute sample size since previously published standard benchmark sets are used.
Data exclusions	No targets were excluded.
Replication	Not applicable. ColabFold is exclusively a computational method. The computional method is deterministic (same result each time you run) when run on the same computer setup. This is why replicates are not needed, as the result would be identical for each replicate.
Randomization	Not applicable. We are not comparing across groups.
Blinding	Not applicable. We are not comparing across groups.

Reporting for specific materials, systems and methods

We require information from authors about some types of materials, experimental systems and methods used in many studies. Here, indicate whether each material, system or method listed is relevant to your study. If you are not sure if a list item applies to your research, read the appropriate section before selecting a response.

Materials & experimental systems

n/a	Involved in the study
☒	☐ Antibodies
☒	☐ Eukaryotic cell lines
☒	☐ Palaeontology and archaeology
☒	☐ Animals and other organisms
☒	☐ Human research participants
☒	☐ Clinical data
☒	☐ Dual use research of concern

Methods

n/a	Involved in the study
☒	☐ ChIP-seq
☒	☐ Flow cytometry
☒	☐ MRI-based neuroimaging
